# Comment on Hoppe et al. Bilateral Symmetrical Brain MRI Findings in Acute Necrotising Encephalopathy Type 1. *Children* 2025, *12*, 974

**DOI:** 10.3390/children12091182

**Published:** 2025-09-04

**Authors:** Memik Teke

**Affiliations:** Department of Radiology, University of Colorado, Aurora, CO 80045, USA; memik.teke@cuanschutz.edu

I read with great interest the recent case report by Alexander T. Hoppe et al. [1], which presents a clinically meaningful case of acute necrotizing encephalopathy type 1. The report highlights an important pediatric neuroimaging case and adds value to the existing literature on this rare condition.

While I found the article informative, I respectfully wish to point out some issues that, if addressed, could further enhance the scientific and educational impact of this case report.

First, the manuscript mentions a “3-month follow-up MRI with complete resolution,” but this image is not included in the main text. Providing the follow-up imaging would allow readers to visually confirm the patient’s recovery.

Second, although the article presents DWI findings, ADC maps are not provided. Diffusion restrictions cannot be reliably confirmed without ADC, as DWI hyperintensity alone may result from T2 shine-through [2]. Including ADC maps would improve diagnostic accuracy and educational value.

I believe that supplementing the case report with follow-up MRI and ADC maps would significantly improve its clarity and scientific value for the readership of *Children*.

## References

[1] Hoppe A.T., Ghia T., Warne R., Shipman P., Lakshmanan R. (2025). Bilateral Symmetrical Brain MRI Findings in Acute Necrotising Encephalopathy Type 1. Children.

[2] Dmytriw A.A., Sawlani V., Shankar J. (2017). Diffusion-Weighted Imaging of the Brain: Beyond Stroke. Can. Assoc. Radiol. J..

